# Quality and Processing Behavior of Egg White and Yolk from Commercial Free-Range and Barn-Laid Eggs: Physical, Compositional and Rheological Assessment in Raw and Heat-Treated (Grilled) States

**DOI:** 10.3390/foods15101682

**Published:** 2026-05-12

**Authors:** María Dolores Álvarez, Victor G. Almendro-Vedia, Beatriz Herranz

**Affiliations:** 1 Institute of Food Science, Technology and Nutrition (ICTAN-CSIC), c/José Antonio Novais, 6, 28040 Madrid, Spain; 2 Department of Food Technology, Faculty of Veterinary, Complutense University, 28040 Madrid, Spain; vgavedia@fis.ucm.es; 3 Pluridisciplinary Institute, Complutense University, 28040 Madrid, Spain

**Keywords:** barn-laid eggs, egg white, egg yolk, fatty acid profile, free-range eggs, griddle cooking, microscopy, rheology, thermal processing

## Abstract

This study evaluated how two commercial egg types (free-range and barn-laid) influence the physical, compositional, and rheological properties of egg white and yolk in raw and grilled states. Free-range eggs showed stronger correlations between external dimensions and internal composition, suggesting potential for nondestructive grading, whereas barn eggs exhibited heavier shells but weaker morphometric–composition relationships. Haugh units differentiated production systems, and yolk redness was the only color parameter clearly associated with free-range origin. Mechanical tests revealed that barn eggs had shells capable of absorbing more energy during rupture. Rheological measurements showed matrix-dependent behaviors: in raw samples, egg white behaved as a weakly structured viscoelastic fluid, while yolk exhibited characteristics of a concentrated lipoprotein emulsion. Stress, frequency, and temperature sweeps revealed contrasting behaviors between the two commercial egg types: barn-laid eggs displayed a stronger egg-white protein network, whereas free-range eggs showed a more reinforced yolk lipoprotein matrix under the conditions evaluated. Yolk behavior fitted the weak gel model with excellent accuracy (*R*^2^ ≈ 1), while egg white did not. Steady shear and three-step tests confirmed pronounced shear thinning and thixotropic behavior in both matrices, with barn eggs showing higher viscosities but lower structural recovery. Thermal treatment reduced the strong rheological differences between raw egg white and yolk, yet production system effects persisted. All grilled samples behaved as weak gels, with barn egg whites forming stiffer networks and free-range yolks generating more elastic, cohesive, and energy-absorbing gels. A trend toward higher MUFA levels was observed in raw free-range yolks. Microscopy further clarified how production system shapes the structural and functional behavior of egg matrices.

## 1. Introduction

Eggs are widely recognized as nutrient-rich and functionally versatile foods. Their balanced composition of high-quality proteins, lipids, vitamins, and minerals makes them a key dietary component across cultures [1,2,3]. Beyond their nutritional relevance, eggs play a central technological role in food processing due to their emulsifying, foaming, gelling, and thermal coagulation properties [4,5,6]. These functionalities arise from the distinct structural organization of egg white and yolk, which respond sensitively to changes in pH, mineral content, lipid composition, and thermal conditions [7,8,9]. Understanding how these matrices behave under different intrinsic and extrinsic factors is therefore essential for predicting egg quality and performance in both culinary and industrial applications. However, the combined analysis of raw and cooked matrices, separately evaluating white and yolk through both microstructural and advanced rheological approaches, remains largely unexplored.

The production system is one of the factors most frequently associated with variability in egg quality. Differences between free-range and barn or caged housing have been reported in internal composition, yolk pigmentation, Haugh units (HU), mineral content and shell characteristics [10,11,12,13]. Free-range eggs often exhibit greater phenotypic variability and higher carotenoid deposition [14,15], whereas barn or caged eggs tend to show more uniformity and increased shell weight or structural consistency [10,12,13]. However, the literature remains inconsistent regarding the magnitude and direction of these effects, partly due to differences in breeds, feeding strategies and analytical approaches [16]. In a food sector increasingly focused on product quality, sustainability and consumer expectations, understanding how production practices shape the structural and functional properties of eggs has become particularly relevant.

Rheology provides a powerful framework for characterizing the structural behavior of egg white and yolk. Egg white is typically described as a weakly structured viscoelastic fluid dominated by a protein network sensitive to ionic strength and thermal denaturation [17], while yolk behaves as a concentrated lipoprotein emulsion with weak gel characteristics, where interactions among low-density lipoproteins (LDL), high-density lipoproteins (HDL) and granule components determine its mechanical response [18,19,20,21]. Rheological measurements—including stress, frequency and temperature sweeps, as well as steady shear and thixotropic tests—offer detailed insights into viscoelasticity, structural resilience, flow behavior and gelation capacity [22]. Despite this potential, few studies have compared white and yolk separately using advanced rheological methods [17,23], and even fewer have examined how these properties differ between production systems. Moreover, when working with commercial eggs, production-related factors such as diet or flock characteristics cannot be isolated from the legally defined housing system, making it difficult to attribute differences to a single cause. This reinforces the need for studies that characterize the technological behavior of eggs as they are available to consumers.

Thermal processing further modifies the structural and functional behavior of egg matrices. Heating induces protein denaturation, aggregation and network formation, leading to profound changes in elasticity, viscosity and mechanical stability [4,6,24,25]. Most studies have focused on raw eggs, whereas the rheology of cooked matrices—particularly when white and yolk are analyzed independently—remains underexplored. This gap is especially relevant for culinary and food-service applications, where texture, structural integrity and flow resistance determine product performance. In this context, griddle cooking represents a thermally controlled and widely used method that produces rapid and reproducible gelation while maintaining white and yolk as separate matrices, making it an ideal model for studying thermally induced gels under realistic culinary conditions.

Despite the importance of these factors, few studies have simultaneously examined morphometric traits, internal composition, yolk lipid profile, color, shell mechanical properties, microstructural features, and advanced rheological behavior to compare free-range and barn eggs in both raw and cooked states. Moreover, the matrix-specific responses of white and yolk to production system and thermal treatment remain insufficiently characterized, as do their implications for technological and culinary performance.

The aim of this study was therefore to assess how commercially available free-range and barn-laid eggs differ in their physical, compositional, microstructural and rheological properties. This study separately characterizes the rheological behavior of egg white and yolk through stress, frequency and temperature sweeps, steady-shear and thixotropic tests, complemented by analyses of lipid profile, morphology and microstructure. The effects of griddle cooking on these properties are also evaluated. By integrating structural, mechanical and nutritional assessments, this work provides a comprehensive understanding of how the production system influences egg quality and its relevance for technological and culinary applications.

## 2. Materials and Methods

### 2.1. Materials

Eggs from two legally defined production systems were used: free-range (code “1”) and barn (code “2”), as established by Commission Regulation (EC) No 589/2008 [26]. All eggs were produced in Spain (“ES” code), had brown shells, and were marketed as category A. Samples belonged to the commercial M/L weight classes (53–73 g) and were purchased in packs of ten from a local supermarket. Ten packages, with 10 eggs by package, per system were acquired, yielding 100 eggs per group. Eggs were stored at 4 °C and analyzed before the “best before” date.

Because this study focuses on the technological characterization of commercial eggs, no information on breed, diet, or farm-level environmental conditions was available, as these parameters are not disclosed on packaging. Production system was therefore taken as declared on the label, reflecting real market conditions. According to Council Directive 1999/74/EC [27], free-range hens have daytime outdoor access, whereas barn hens are housed in floor-based barn systems.

On the other hand, because the eggs were purchased as retail products, information on the exact number of days from lay was not available; therefore, egg freshness could not be controlled and may have contributed to variability in egg white-related measurements.

### 2.2. Sample Preparation

Egg white and yolk were analyzed separately in both raw and grilled eggs. Raw egg white from free-range and barn-laid eggs was designated RW1 and RW2, respectively, while raw yolk was designated RY1 and RY2. Grilled egg white and yolk were labeled GW1 and GW2, and GY1 and GY2.

Grilled samples were prepared using a grill plate (JT950, 2500 W; Electrodomésticos Jata, Tudela, Navarra, Spain) set to level 3 (160 °C). The center of the plate was lightly greased with olive pomace oil. Each whole egg (unmodified, with its natural dimensions) was cracked directly onto the plate and cooked for 4.25 min, during which the yolk′s thermal center briefly reached 65 °C. This temperature was verified in every sample using a Lab Thermometer IP65 LT 101 (TFA Dostmann, Wertheim, Germany). After cooking, samples were allowed to cool to room temperature and were immediately processed for subsequent analyses.

### 2.3. Physical Measurements (Weights and Dimensions)

The weights of the whole egg, yolk, white, and shell were recorded using an analytical balance (AU 220 4M; KERN & SOHN GmbH, Balingen, Germany). Egg dimensions (equatorial diameter and height) were measured with a digital caliper. Measurements were performed on 20 eggs per rearing system, selecting two eggs from each of the ten purchased packages.

Each egg was opened manually, and yolk and egg white were separated following the procedure of Harrison and Cunningham [28]. The intact yolk, still enclosed in the vitelline membrane, was placed on absorbent paper to remove residual thick egg white. The membrane was then punctured, allowing the yolk to drain into a pre-weighed weigh boat while avoiding inclusion of egg white or chalazae. The entire egg white was transferred into a separate pre-weighed container. Eggshells were rinsed to remove egg white residues, membranes were carefully removed, and shells were gently dried with absorbent paper before being weighed in pre-weighed dishes.

### 2.4. Haugh Units (HU)

The height of the thick egg white was measured using a QCH gauge (TSS, York, UK), positioning the probe at the highest point of the dense egg white immediately after breaking the egg. HU were calculated only for eggs in which a reliable measurement of egg white height could be obtained (twenty measurements per rearing systems). HU were computed according to the standard expression (Equation (1)):

(1) *HU* = 100 ⋅ log (*h* − 1.7 ⋅ *w*^0.37^ + 7.6)

where *h* is the thick egg white height (mm) and *w* is the whole egg weight (g) [29,30].

### 2.5. Color of Raw Yolk Eggs

Yolk color of six eggs was measured on a glass plate using a Konica Minolta CM-3500D spectrophotometer (Konica Minolta Business Technologies, Tokyo, Japan) configured with a D65 illuminant and a 10° standard observer. The CIELAB coordinates L*, a*, and b* were recorded.

### 2.6. Mechanical Characterization of Eggshell Rupture

Shell rupture properties were evaluated using a TA.XT2i Texture Analyzer (Stable Micro Systems, Godalming, UK) equipped with a 30 kg load cell, following the compression protocol described by Herranz et al. [29]. Eggs were placed horizontally on a three-point support and compressed with a 45 mm flat cylindrical probe at 1 mm/s (trigger force: 0.001 N). Force–time data were collected at 500 pps. From the resulting curves, shell breaking force (N), rupture area (N·s), and the number of rupture peaks (force drops > 0.05 N) were obtained. Tests were performed on twenty eggs per rearing system.

### 2.7. Rheological Measurements

Rheological measurements of raw and grilled egg white and yolk were performed using a controlled-stress rheometer (Kinexus Pro; NETZSCH Gerätebau GmbH, Selb, Germany). Raw egg white was analyzed using a concentric cylinder geometry with a stainless-steel bob (C25, 25 mm) and a PC25 C0155 AL cup mounted on a Peltier cylinder cartridge (sample volume: 17.61 mL; bob gap: 9.150 mm).

Raw yolk was measured using a cone-and-plate geometry (CP1/40; 1° angle, 40 mm diameter) mounted on a Peltier plate cartridge (sample volume: 0.29 mL; gap: 0.0316 mm). Grilled egg white was tested using a roughened plate–plate geometry (PU20) with a 2 mm gap. Samples were extracted with a 20 mm corn borer to obtain discs matching the plate diameter.

Grilled yolk exhibited a heterogeneous structure, with partially fluid areas interspersed with more coagulated regions. Because this prevented the extraction of uniform discs, the yolk was collected manually with a laboratory spoon and analyzed using a smooth plate–plate geometry (PU20) with a 1 mm gap. All plate geometries were temperature-controlled with a Peltier cartridge, and samples were covered with a plastic solvent trap to minimize evaporation.

Oscillatory shear measurements of raw white and yolk were conducted at 5 °C, corresponding to typical refrigerated storage conditions, while steady-shear measurements were performed at 20 °C to reflect ambient handling conditions. Rheological measurements of grilled white and yolk were carried out at 25 °C, as higher temperatures promote cooking and drying phenomena. At least three biological replicates were performed for each treatment group, using three different eggs, each providing its own white and corresponding yolk. Every rheological measurement was therefore carried out on a distinct egg. No technical replicates were generated because each rheological assay requires a complete measurement sequence per sample and is highly time-consuming, particularly for raw egg white due to the low oscillatory frequency (0.1 Hz) needed for reliable measurements.

#### 2.7.1. Small Amplitude Oscillatory Shear (SAOS) Measurements

1. Time Sweep Tests

All raw samples (RW1, RW2, RY1, and RY2) were loaded onto the corresponding geometry and allowed to rest for 5 min. Time sweep tests were then performed for 20 min to allow stabilization and structural reorganization after loading. Measurements were conducted at 0.1 Hz for raw egg white and 1 Hz for raw yolk, using stresses within the linear viscoelastic region (LVR): 0.01 Pa for RW1, 0.1 Pa for RW2, and 0.5 Pa for RY1 and RY2. Grilled samples (GW1, GW2, GY1, and GY2) were also allowed to rest for 5 min on the geometry to stabilize and reach thermal equilibrium, but no time sweeps were performed.

2. Stress Sweep Tests

The LVR of each sample was determined by stress sweep tests. A constant frequency of 0.1 Hz was used for RW1 and RW2, and 1 Hz for RY1 and RY2. The applied shear stress ranged from 0.001 to 0.1 Pa for RW1, 0.01 to 1 Pa for RW2, and 0.05 to 5 Pa for RY1 and RY2. For grilled samples (GW1, GW2, GY1, and GY2), the frequency was fixed at 1 Hz and the stress range was 0.1–1000 Pa. A total of 41 data points was collected per test. The storage modulus *G*′, loss modulus *G*″, and complex modulus G* (calculated as $G^{*}=\;\sqrt{{G\prime }^{2}+{G\prime \prime }^{2}}$) were monitored throughout the tests. Critical stress *σ*_max_ and strain *γ*_max_ were determined from the evolution of G*, applying a tolerance of ±10% for all samples [30,31,32] except for RW1, for which a stricter tolerance of ±5% was used. Differences in the LVR of raw and grilled samples were further evaluated by fitting a linear regression between stress *σ* and strain *γ* for the G* data, from the initial values (*σ*_0_, *γ*_0_) to (*σ*_max_, *γ*_max_) (Equation (2)):

(2) *σ*_complex_ = *a* · *γ* + *b*

The slope *a* (Pa) reflects the overall resistance to deformation, including both elastic and viscous contributions, and is considered equivalent to gel strength [31,32]. The intercept *b* (Pa) represents the initial stress *σ*_0_ at *γ*_0_. Both parameters were used to calculate the energy term *E* (Equation (3)), which quantifies the total energy involved in the linear deformation or toughness [30,33] of the white and yolk network:

(3) $$E=\;\int_{\gamma_{0}}^{\gamma_{max}}\left(a\cdot \gamma +b\right)\cdot d\gamma$$

3. Frequency Sweep Tests

Frequency sweeps were performed by applying harmonic strain oscillations at fixed stress within the LVR: 0.01 Pa for RW1, 0.1 Pa for RW2, 0.5 Pa for RY1 and RY2, and 1 Pa for all grilled samples (GW1, GW2, GY1, and GY2). The frequency range was 0.01–1 Hz for RW1 and RW2, and 0.1–10 Hz for the remaining samples. The parameters *G*′, *G*″, loss factor tan *δ* = *G*″/*G*′, and complex viscosity *η*∗ = G*/*ω* (where *ω* is the angular frequency in rad/s) were obtained as functions of frequency.

Except for RW1 and RW2, G* was also fitted to the weak gel model [34] (Equation (4)):

(4) G*(*f*) = *Af*^1/z^

where G* is the complex modulus (Pa), f is the frequency (Hz), *A* (Pa·s^1/z^) is the proportionality constant representing interaction strength (i.e., G* at 1 Hz), and *z* (dimensionless) is the coordination number or network connectivity [2,35,36], considered an indicator of structural organization.

4. Temperature Sweep Tests

Temperature sweeps were carried out from 5 to 50 °C at a heating rate of 5 °C/min. Measurements were performed at 0.1 Hz for raw egg white and 1 Hz for raw yolk, using stress values previously selected within the LVR for each sample. The evolution of *G*′, *G*″, and phase angle *δ* was recorded as a function of temperature.

#### 2.7.2. Steady Shear Measurements

1. Flow Behavior

All raw and grilled white and yolk samples were allowed to rest on the geometry for 5 min to stabilize and reach thermal equilibrium, and were then pre-sheared at 100 s^−1^ for 1 min. Flow curves were obtained by decreasing the shear rate from 100 to 0.01 s^−1^, with five measurement points per decade.

The dependence of shear stress on shear rate was described by the power-law model (Equation (5)):

(5) $$\sigma \left(\dot{\gamma }\right)=K{\dot{\gamma }}^{n}$$

where *σ* is the shear stress (Pa), $\dot{\gamma }$ is the shear rate (s^−1^), *K* is the consistency index (Pa·s^n^), corresponding to the apparent viscosity at 1 s^−1^, and *n* is the flow behavior index (dimensionless), indicating the deviation from Newtonian behavior. Apparent viscosities at 50 s^−1^ (referenced by the National Dysphagia Diet [37]), 10 s^−1^ (oral shear conditions; [38]), and 0.1 s^−1^ (low-shear processes) were also evaluated for all samples.

2. Three-Step Shear Rate Tests

For viscometric rebuild analysis, raw white and yolk samples were first subjected to a shear rate of 0.1 s^−1^ for 30 s to obtain the initial apparent viscosity (*η*_0_). The shear rate was then increased to 100 s^−1^ for 30 s to induce structural breakdown, and finally reduced again to 0.1 s^−1^, monitoring viscosity recovery for 600 s. The final apparent viscosity at the end of this recovery period (*η*_f_) was used to calculate the percentage of viscosity recovery as (*η*_f_ × 100)/*η*_0_ [39].

### 2.8. Fatty Acids (FAs) Profile

The fatty acid (FA) profile of raw yolks from free-range and barn systems was determined following a saponification and bimethylation procedure using C13:0 as internal standard, adapted from Álvarez et al. [40]. Fatty acid methyl esters (FAMEs) were analyzed by GC-FID using a polar capillary column suitable for FA separation. Identification was performed by comparison of retention times with three commercial reference mixtures: FAME 37 Mix (Supelco, Bellefonte, PA, USA; Ref. CRM47885), PUFA No. 2 Animal Source (Sigma-Aldrich, St. Louis, MO, USA; Ref. 47015‑U), and PUFA No. 3 Menhaden Oil (Sigma, Ref. 47085-U). Results were expressed as mg FA/g yolk. Lipid profile analyses were performed in triplicate on freeze-dried yolks.

### 2.9. Brightfield and Epifluorescence Microscopy

Micrographs for microscopy of raw and grilled egg whites and yolks were obtained following a procedure adapted from Herranz et al. [30]. For whites, a small portion of each sample was placed on a glass slide, gently flattened with a spatula, and covered with a coverslip. For the yolk samples, a micropipette tip was inserted into the yolk and the contents were gently mixed. To facilitate sample extraction, the tip was cut and 50 µL of yolk were collected and deposited onto a coverslide, then covered with a coverslip. Once the drop was completely spread, it was observed under the microscope.

Images were acquired using an inverted microscope (Leica DMi8Leica Microsystems, Wetzlar, Germany) equipped for brightfield and epifluorescence imaging, fitted with a digital camera (sCMOS monochrome digital camera Leica K5, 1 ms acquisition time per channel) and standard acquisition software (Leica Application Suite X—LAS X; Leica Microsystems, Wetzlar, Germany). The system included LED illumination (CoolLED pE300 lite SB; CoolLED Ltd., Andover, UK) and appropriate filter sets for routine structural observation (Ex/Em [nm]: 405/60, 470/40; 480/40, 527/30; 546/10, 585/40; 620/60, 700/75). A 10× AN 0.25, WD 12 mm plan-achromatic objective was used for all micrographs.

### 2.10. Statistical Analysis

Statistical comparisons between egg production systems and cooking conditions were performed using two-tailed *t* tests for independent samples (*α* = 0.05). Because egg white and yolk represent fundamentally different physicochemical systems, and raw and grilled samples undergo distinct structural transformations, statistical comparisons were performed within each matrix × processing stratum, where the production system was the only meaningful fixed factor. Because commercial packages did not represent biologically meaningful batches (e.g., flock or farm), no random batch effect could be defined, and each egg was treated as an independent experimental unit. Variance homogeneity was evaluated for each comparison: Student′s *t* test with pooled variance was applied when variances were comparable, whereas Welch′s *t* test was used when unequal variances or unbalanced sample sizes were detected. Results are reported as the mean ± standard deviation (SD), and statistical significance was set at *p* < 0.05. No correction for multiple testing was applied, as the study was exploratory in nature; accordingly, the risk of Type I error is increased, and marginal *p*-values should be interpreted with caution. Data were analyzed using SPSS for Windows, version 29.0.0.0 (IBM SPSS Statistics, Armonk, NY, USA).

## 3. Results and Discussion

### 3.1. Physical Measurements (Weights and Dimensions) in Raw Eggs

Eggs from barn-kept hens tended to be heavier than those from free-range hens (Table 1), although only egg white and shell weights differed significantly (*p* = 0.021 and *p* = 0.024). Whole-egg and yolk masses showed no statistical differences (*p* = 0.055 and *p* = 0.641), and external dimensions were similar between systems (equatorial diameter: *p* = 0.231; egg height: *p* = 0.627). Because whole egg weight did not differ between production systems, this variable cannot account for the differences observed in internal traits and does not function as a meaningful covariate in this dataset. Free-range eggs exhibited greater variability in most traits, consistent with the heterogeneous environmental conditions typical of outdoor production. Comparable patterns have been reported by Gałązka-Czarnecka et al. [11] and by Marelli et al. [13], who also observed higher variability in free-range systems.

Correlation analysis revealed that morphometric traits were more strongly associated with internal composition in free-range eggs than in barn eggs (Table 2). In free-range eggs, whole-egg weight correlated strongly with egg white mass (*r* = 0.907, *p* < 0.01) and equatorial diameter (*r* = 0.818, *p* < 0.01), while egg height was strongly associated with (*r* = 0.760, *p* < 0.01). These associations reflect the general principle that external dimensions capture part of the internal volume distribution. Similar trends have been described in eggs produced under variable environmental conditions [10,11,12].

In barn eggs, correlations were only moderate—between whole-egg weight and egg white mass (*r* = 0.704, *p* < 0.05) and between whole-egg weight and equatorial diameter (*r* = 0.684, *p* < 0.05)—and relationships involving yolk mass were not significant. This weaker connectivity is consistent with previous studies reporting that barn production systems, characterized by standardized diets and controlled microenvironments, exhibit lower phenotypic variability, which in turn reduces the strength of correlations between external and internal egg traits [13,16]. Marelli et al. [13] specifically showed that barn systems produce more homogeneous eggs than free-range systems, while Roberts [16] highlighted that environmental and nutritional uniformity reduces variability in egg quality parameters.

### 3.2. Haugh Units (HU) of Raw Eggs

HU differed significantly between production systems. Free-range eggs showed higher internal quality (63.40 ± 3.76) than barn eggs (55.06 ± 3.46; *p* = 0.010). Although both were marketed as Category A according to EU standards—which assess only external quality—the HU values obtained here are consistent with previous studies reporting higher egg white quality in free-range or alternative systems compared with barn or cage housing [10,11,13]. However, not all comparisons among non-cage systems yield the same pattern; for example, organic eggs have been reported to show lower HU than cage eggs in some contexts [41]. These results indicate that free-range eggs exhibited superior egg white quality under the conditions evaluated.

### 3.3. Color of Raw Egg Yolks

The color parameters of raw egg yolks differed between production systems. Free-range eggs showed slightly higher lightness (L) and yellowness (b) values than barn eggs, although neither difference was statistically significant. In contrast, redness (a*) was markedly higher in free-range yolks (17.81 ± 0.91 vs. 6.69 ± 0.70; *p* < 0.0001), which is consistent with substantially greater deposition of red carotenoids. This pattern is consistent with the higher availability of natural pigment sources—such as green vegetation, insects, and plant-derived xanthophylls—in outdoor systems. Dietary carotenoids are well-established determinants of yolk pigmentation intensity, and both reviews and experimental studies have shown that increased intake of lutein, zeaxanthin and other plant pigments leads to higher a* and b* values in egg yolks [14,15]. These findings are compatible with the interpretation that the enhanced redness observed in free-range yolks may reflect greater access to natural carotenoid sources.

### 3.4. Mechanical Characterization of Eggshell Rupture

**Figure 1:**
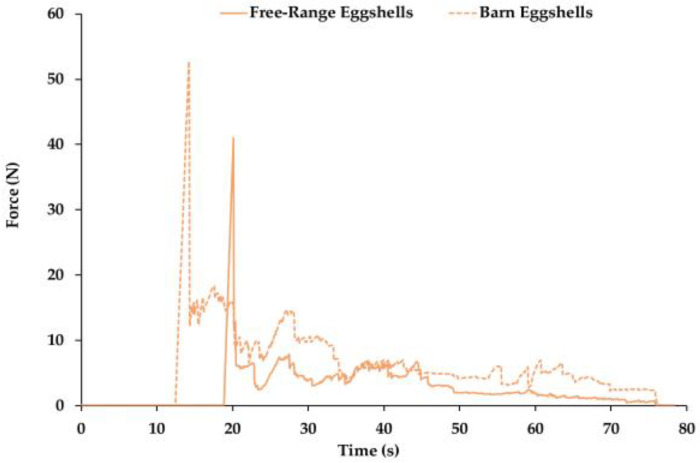
Representative force–time curves obtained during the instrumental shell rupture test for free-range and barn eggs.

Representative force–time curves for free-range and barn eggs are shown in Figure 1. Because data acquisition begins once the trigger force (0.001 N) is detected, the curves do not start at the origin; the first recorded point corresponds to the moment of effective contact.

Both egg types exhibited a clear maximum force peak associated with shell-breaking followed by multiple smaller fracture peaks until complete rupture at approximately 80 s, consistent with previous observations in ISA White eggs [29]. No significant differences were found between commercial types in shell breaking force (free-range: 47.97 ± 5.48 N; barn-laid: 48.36 ± 3.48 N; *p* = 0.89) or in the number of fracture peaks (*p* = 0.17). However, the total rupture area differed markedly: barn eggs required more energy to fracture (356.68 ± 33.50 N·s) than free-range eggs (282.30 ± 22.60 N·s; *p* = 0.003).

This greater energy absorption was associated with their higher shell weight (Table 1), as increased mineral deposition and thicker calcified layers are known to contribute to the shell′s capacity to dissipate energy during fracture. According to Nys et al. [42], the palisade layer and the degree of calcite crystal organization play a central role in determining shell toughness, with more densely mineralized structures exhibiting improved resistance to crack propagation. The shell breaking forces obtained here were slightly higher than those reported for hens fed grape pomace (45.6 ± 1.50 N), whereas the rupture areas in that study were larger (413 ± 15.5 N·s).

Overall, despite similar maximum forces, barn eggs showed a shell structure compatible with absorbing more energy during rupture. However, because factors such as mineral intake, hen physiology, and flock management were not controlled in this study, these differences should be interpreted as associations within the commercial samples analyzed, rather than direct effects of the production system. Given the test speed (1 mm/s), the area under the force–time curve corresponds directly to energy (J), allowing comparison of fracture resistance between samples.

### 3.5. SAOS Measurements of Raw Egg Whites and Yolks

#### 3.5.1. Stress Sweep Tests

**Figure 2:**
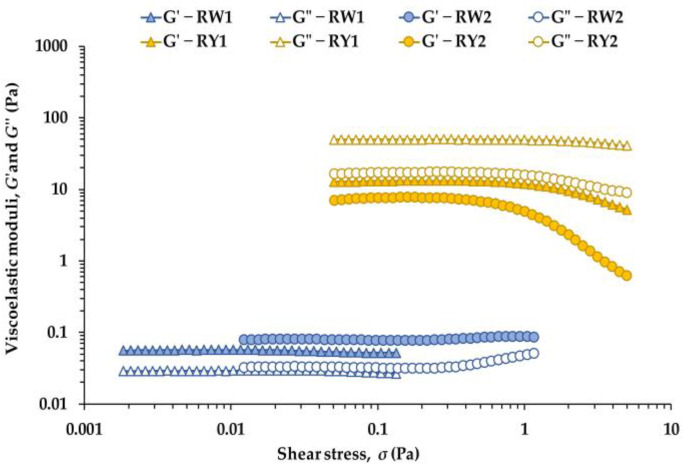
Storage (*G*′) and loss (*G*″) moduli of raw egg white (RW1 = free-range, RW2 = barn-laid) and yolk (RY1 = free-range, RY2 = barn-laid) as a function of shear stress at 5 °C. The linear viscoelastic region (LVR) corresponds to the plateau of *G*′ before its deviation at the critical stress (*σ*_max_).

Figure 2 shows the linear viscoelastic region (LVR) of raw egg white and yolk at 5 °C for both production systems. Marked differences between matrices were evident both within the LVR and at its boundary. Egg white samples (RW1, RW2) exhibited a predominantly solid-like response, with *G*′ consistently higher than *G*″, whereas yolk samples (RY1, RY2) showed the opposite trend, with *G*″ > *G*′ throughout the entire stress range. These contrasting signatures reflect the distinct microstructures of egg white and yolk.

In egg white, the solid-like behavior is consistent with its protein-based network, composed mainly of ovalbumin, ovotransferrin, ovomucoid and, particularly, the filamentous glycoprotein ovomucin [1,8,43]. Ovomucin aggregates contribute to a weakly connected viscoelastic network that yields *G*′ > *G*″ at small deformations, although this structure does not meet the criteria of a true weak gel according to classical weak-gel theory [34]. Egg white is therefore better described as a weakly structured viscoelastic fluid or protein-based suspension [17,25,30,44], whose viscoelastic properties are highly sensitive to intrinsic factors such as genetic line and to storage conditions [30].

Yolk samples, in contrast, displayed a liquid-like response dominated by viscous dissipation. Their much higher moduli and *G*″ > *G*′ behavior are consistent with the rheology of concentrated oil-in-water emulsions stabilized by lipoproteins. Yolk contains ~65% lipids and ~30% proteins organized into plasma (rich in low-density lipoproteins, LDL) and granules (rich in high-density lipoproteins, HDL, and phosvitin) [9,45]. This supramolecular structure is compatible with a dense emulsion whose viscoelasticity is influenced by lipoprotein interactions and energy dissipation [19,21]. Once *σ*_max_ is reached, both G′ and G″ decrease, consistent with structural breakdown of the lipoprotein matrix, a typical response of concentrated emulsions [45].

Differences between production systems were also evident. RW2 exhibited higher *G*′ and *G*″ values and a higher *σ*_max_ than RW1, consistent with a stronger and more cohesive protein network (Figure 2). Conversely, yolk samples showed the opposite trend: RY1 (free-range) displayed higher moduli than RY2, compatible with a denser lipoprotein matrix in free-range yolks. The slight increase in *G*′ and *G*″ observed in RW2 at *σ*_max_, and the less clearly defined LVR in RW1, explain the stricter ±5% tolerance required for RW1.

Table 3 summarizes the critical rheological parameters at the end of the LVR. In egg whites, RW2 reached significantly higher *σ*_max_ and *γ*_max_ values than RW1 (*p* < 0.0001), indicating a more deformation-resistant and extensible protein network. RW2 also showed a higher critical tan *δ* (*p* = 0.014), indicating a slightly more dissipative viscoelastic balance at the onset of nonlinearity. In yolks, RY1 exhibited the highest *σ*_max_ (*p* < 0.0001), whereas RY2 showed a significantly higher *γ*_max_ (*p* = 0.041), consistent with differences in the connectivity and resistance of their lipoprotein matrices. As expected, yolks displayed much higher tan *δ* values than whites, reflecting their predominantly viscous character.

**Figure 3:**
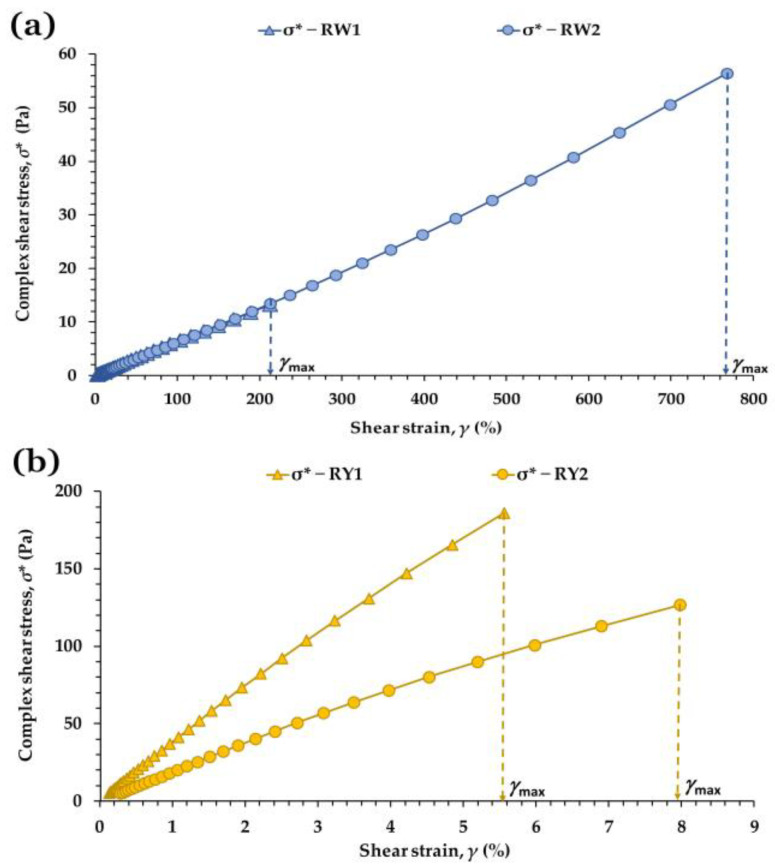
Complex shear stress (σ*) as a function of strain (*γ*) for raw egg white (RW1, RW2) and yolk (RY1, RY2) at 5 °C. (**a**) Egg white samples. (**b**) Egg yolk samples.

To further characterize the transition from linear to nonlinear behavior, the evolution of complex shear stress (*σ*) with strain (*γ*) was examined (Figure 3). This representation provides complementary insight into whether nonlinearity arises from structural softening, strain-induced strengthening, or other matrix-specific mechanisms. Linear regressions applied to the initial segment of each σ*–*γ* curve yielded the coefficients *a* and *b* (Equation (1)), which quantify the stiffness at very small deformations and the effective stress at the origin, respectively. This approach aligns with the framework proposed by Fiszman and Tovar [33], who emphasize the relevance of small-amplitude rheology for understanding intrinsic gel structure.

In egg whites, RW2 showed a significantly higher *a* value than RW1 (*p* < 0.05), consistent with its higher *σ*_max_ and *γ*_max_ (Table 3). The *b* parameter also differed markedly (*p* < 0.001): RW1 exhibited a small positive intercept (0.078 Pa), whereas RW2 showed a negative one (−0.831 Pa). This contrast is compatible with RW2 possessing a more homogeneous and continuous microstructure requiring a small deformation range before the linear response is fully established, while RW1 exhibits immediate resistance at the onset of deformation, consistent with a less uniform protein network [30,43,44].

In yolks, the much larger *a* values reflect the intrinsic rigidity of concentrated lipoprotein emulsions [21,45]. RY1 showed a significantly higher *a* than RY2 (*p* = 0.0035), consistent with a more resistant but less extensible matrix (Table 3). The *b* values did not differ significantly (*p* > 0.05), and both remained positive, suggesting that the initial response in both yolks is dominated by immediate resistance of the lipoprotein network [9,45].

The total energy stored within the LVR (*E*) provides an integrated measure of structural robustness [30,31,32,33]. In egg whites, *E* differed dramatically between samples: RW2 exhibited an energy value almost 17-fold higher than RW1 (*p* < 0.0001), indicating that its protein network is not only stiffer and more resistant but also capable of sustaining substantially larger deformations before leaving the linear regime. This is visually reflected in the larger area under the σ*–*γ* curve of RW2 (Figure 3a). In yolks, *E* did not differ significantly between RY1 and RY2 (*p* = 0.754), indicating that the higher stiffness and lower extensibility of RY1 are compensated by the lower stiffness and higher extensibility of RY2 (Figure 3b). As a result, both yolks store a comparable amount of energy within the LVR, consistent with the deformation mechanisms of concentrated lipoprotein emulsions.

#### 3.5.2. Frequency Sweep Tests

**Figure 4:**
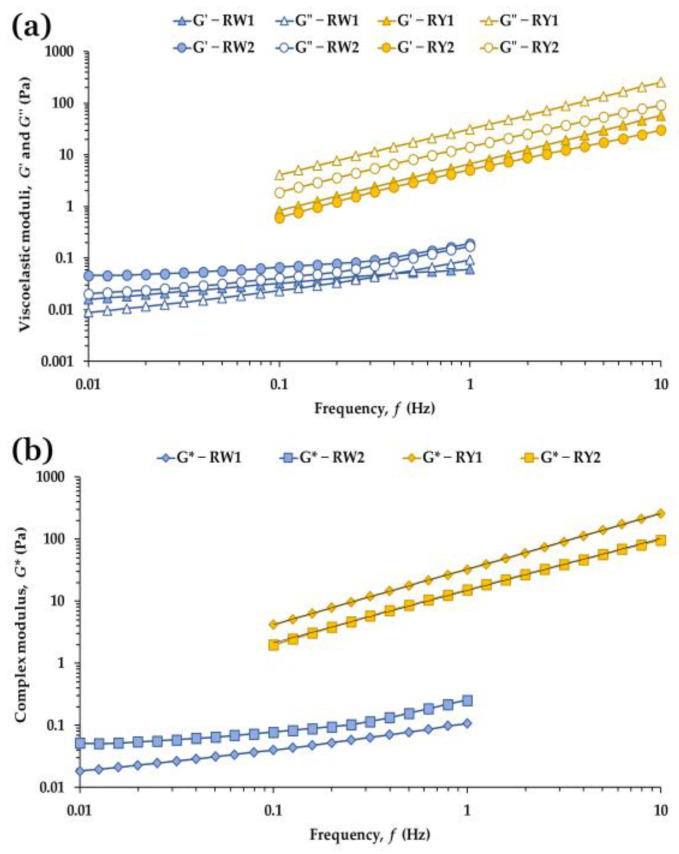
Mechanical spectra of raw egg white (RW1, RW2) and yolk (RY1, RY2) at 5 °C. (**a**) Storage (*G*′) and loss (*G*″) moduli vs. frequency. (**b**) Complex modulus (G*) vs. frequency.

Figure 4 shows the mechanical spectra of raw egg white and yolk at 5 °C for both production systems. In egg whites (Figure 4a), *G*′ remained higher than *G*″ across most of the frequency range, indicating an elastic-dominated response at low and intermediate frequencies. However, the moduli did not follow a common power-law exponent and tan δ varied with frequency. At high frequencies, particularly in RW1, *G*′ and *G*″ converged and eventually crossed, with *G*″ becoming slightly higher than *G*′. This inversion is consistent with the egg white network being unable to sustain elastic stresses under rapid deformation, a behavior characteristic of weakly connected protein matrices whose intermolecular interactions reorganize faster than the imposed oscillation [43,44]. These features support the view that raw egg white does not meet the criteria of a weak gel and behaves instead as a weakly structured viscoelastic fluid, consistent with previous observations for native thick and thin egg white fractions [1,17,30].

Yolk samples displayed the opposite trend: *G*″ exceeded *G*′ throughout the entire frequency range (Figure 4a), consistent with the dominance of viscous dissipation in concentrated lipoprotein emulsions. Despite this liquid-like signature, both moduli increased with frequency following similar power-law exponents, and the weak-gel model provided excellent fits (*R*^2^ ≈ 1; Table 4). This behavior is compatible with very weak gels or transitional viscoelastic systems [46], reflecting the supramolecular organization of yolk plasma and granules, where LDL, HDL and phosphoproteins are organized into a densely packed colloidal matrix [9,45,47].

The production system exerted clear and statistically significant effects on both matrices (Table 4). In egg whites, RW2 exhibited significantly higher *G*′ and *G*″ values than RW1 (*p* < 0.0001), consistent with a more cohesive and concentrated protein network. Both samples showed *G*′ > *G*″ at low frequencies, but their high-frequency behavior differed: RW2 displayed convergence of the moduli, whereas RW1 showed a crossover where *G*″ exceeded *G*′. This shift toward more dissipative dynamics in RW1 is compatible with a less stable microstructure under rapid oscillations. Consistent with these observations, the weak-gel model could not be applied to egg white: the moduli lacked a common exponent, tan *δ* was frequency-dependent, and the structural parameters *A* and *z* could not be reliably determined. These results align with the viscoelastic profile of native egg white and its sensitivity to protein composition, pH and storage conditions [17,25,30,44].

In yolks (Table 4), free-range samples (RY1) exhibited significantly higher *G*′ (*p* = 0.0016), *G*″ (*p* = 0.0002), η* (*p* = 0.0002), tan *δ* (*p* = 0.0006), and weak-gel strength parameter *A* (*p* < 0.0001) than barn-laid yolks (RY2), consistent with a denser and more dissipative lipoprotein matrix. RY1 also showed a lower *z* value (*p* = 0.0048), suggesting reduced frequency dependence and a more stable microstructure. This interpretation is consistent with the conceptual framework described by Di Mattia et al. [2], who reported that lower *z* exponents in yolk-based systems may reflect a smaller number of flow units interacting more strongly with one another, leading to more cohesive and densely connected structures. Although yolk remains a predominantly viscous matrix, such reductions in *z* are consistent with enhanced interparticle interactions within the lipoprotein network, particularly among LDL-rich plasma components and granule-associated proteins [9,45]. Xu et al. [9] showed that stronger LDL–protein associations and tighter packing within the supramolecular structure reduce flowability and increase resistance to deformation, while Anton [45] describes how the organization of plasma lipoproteins and granule proteins governs the viscoelastic response of yolk systems. In this context, the lower *z* of RY1 supports the interpretation that free-range yolks possess a more tightly organized supramolecular structure, consistent with their higher moduli and greater weak-gel strength parameter *A*.

The compositional profiles of egg white and yolk provide a coherent explanation for these rheological differences. In egg white, higher *G*′, *G*″ and η* values in RW2 are consistent with a more concentrated and structurally cohesive protein network, consistent with reports that barn housing can influence egg white quality, protein content and thick-white height [10,11]. Variations in pH and ionic strength may also modulate protein–protein interactions and the extent of the ovomucin–lysozyme complex [17,30,44]. Conversely, the lower stiffness and higher tan *δ* of RW1 are compatible with a more diluted or less interconnected protein matrix, in agreement with previous observations for free-range egg white [12,13].

Similarly, the higher moduli, viscosity and *A* values observed in RY1 are consistent with compositional differences in the yolk lipoprotein matrix (Figure 4; Table 4). Free-range yolks often contain higher levels of lipids, phospholipids and carotenoids [15,29], which enhance viscous dissipation and network cohesion. The lower z exponent in RY1 is compatible with stronger interparticle interactions, consistent with denser LDL aggregates and a more cohesive lipoprotein structure [9,45,47]. In contrast, the lower mechanical strength and higher frequency dependence of RY2 align with a less concentrated or less interconnected lipoprotein matrix, as previously described for yolks from barn housing systems [11,41].

#### 3.5.3. Temperature Sweep Tests

**Figure 5:**
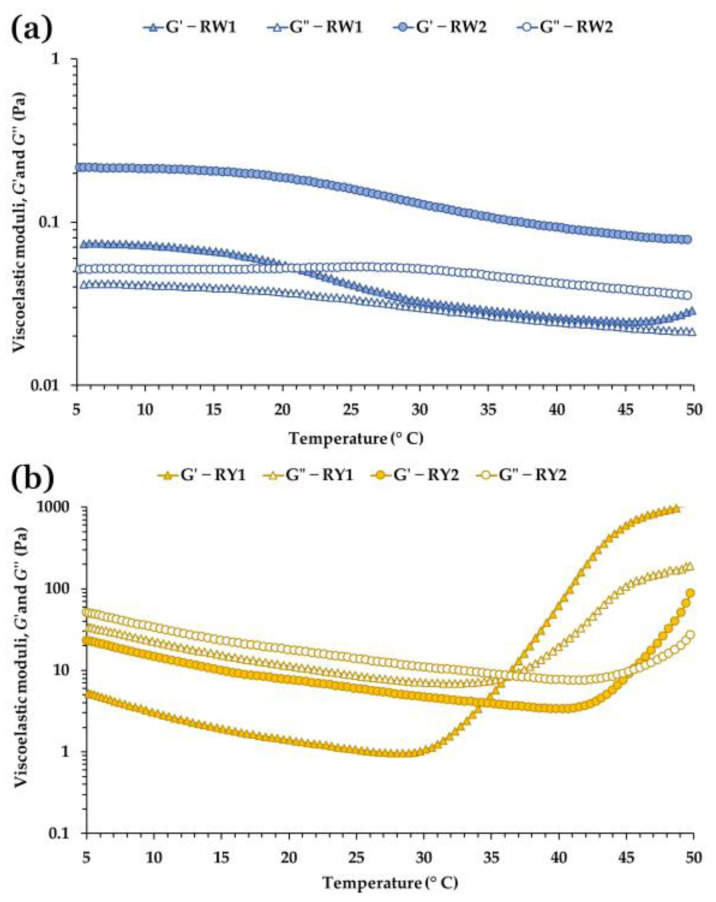
Temperature dependence of the viscoelastic moduli of raw egg white (RW1 = free-range, RW2 = barn-laid) and yolk (RY1 = free-range, RY2 = barn-laid). (**a**) Storage (*G*′) and loss (*G*″) moduli of egg white samples. (**b**) Storage (*G*′) and loss (*G*″) moduli of egg yolk samples.

Temperature sweep tests revealed clear differences between egg white and yolk, as well as significant effects of the production system on their thermal behavior (Figure 5, Table 5). Raw egg whites did not exhibit a detectable crossover temperature within the 5–50 °C range, consistent with their behavior as weakly structured viscoelastic fluids lacking a percolated network [1,4,25]. In both RW1 and RW2, *G*′ and *G*″ decreased progressively with increasing temperature, particularly above 25 °C. This thermal softening is consistent with the progressive unfolding of ovalbumin, ovotransferrin and other egg white proteins, which may weaken the underlying viscoelastic network [17,30,44]. In RW1, the two moduli approached each other closely between approximately 30 and 50 °C, a convergence not observed in RW2 (Figure 5a). At 25 °C, RW2 showed significantly higher *G*′ and *G*″ values than RW1 (*p* < 0.0001 and *p* = 0.0027, respectively; Table 5), consistent with a more cohesive and mechanically resistant protein matrix at ambient temperature. The absence of a crossover in egg white within this temperature range is consistent with the known thermal behavior of egg-white proteins, which do not form a percolated gel network until temperatures exceed ~60 °C, when heat-induced aggregation begins [48].

In contrast, raw egg yolks exhibited a clear thermal transition characterized by a crossover temperature at which *G*′ and *G*″ became equal (Figure 5b). This observation aligns with recent work describing yolk as a model critical gel [49], where the crossover is compatible with temperature-dependent restructuring near the sol–gel transition. RY1 showed a significantly lower crossover temperature (36.8 °C) than RY2 (44.0 °C; *p* = 0.004; Table 5), suggesting earlier strengthening of the elastic component. At 25 °C, both *G*′ and *G*″ were markedly higher in RY2 than in RY1 (*p* < 0.0001 for *G*′; *p* = 0.007 for *G*″), consistent with a more rigid and viscous lipoprotein network in barn-laid yolks.

### 3.6. Steady Shear Measurements of Raw Egg Whites and Yolks

#### 3.6.1. Flow Behavior

**Figure 6:**
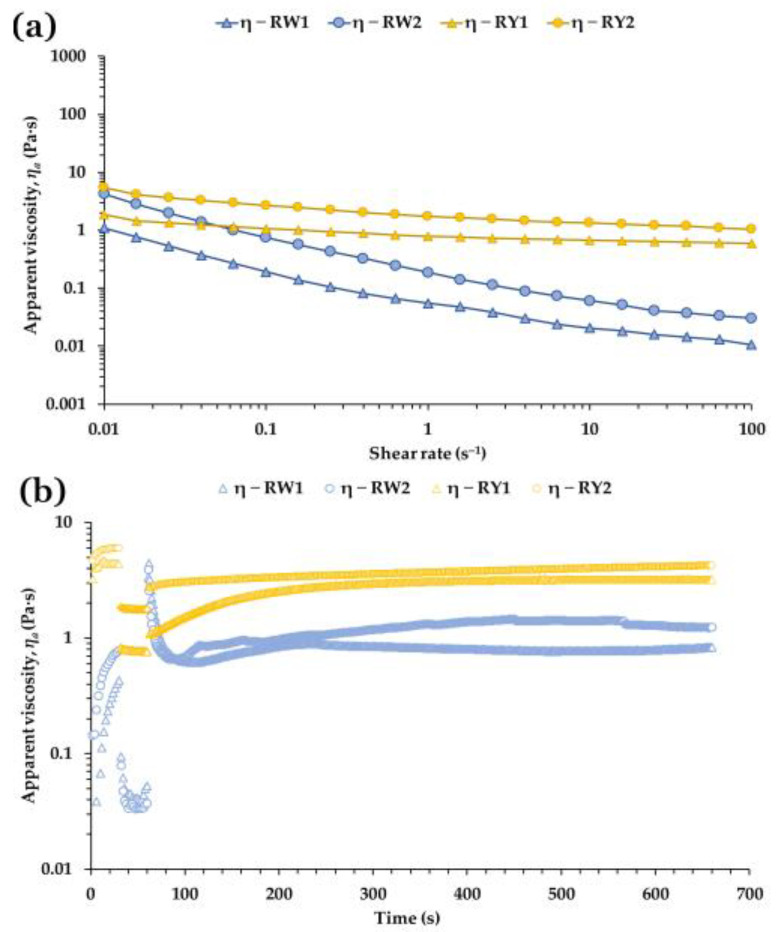
Apparent viscosity of raw egg white (RW1 = free-range, RW2 = barn-laid) and yolk (RY1 = free-range, RY2 = barn-laid) at 20 °C. (**a**) Apparent viscosity as a function of shear rate. (**b**) Apparent viscosity as a function of time during a three-step shear rate test.

Figure 6a shows the flow curves of raw egg white and yolk from free-range and barn eggs measured at 20 °C. All samples exhibited clear shear-thinning behavior, with apparent viscosity decreasing as shear rate increased. This response is characteristic of protein-rich dispersions and weakly structured emulsions, where intermolecular interactions may be progressively weakened under flow [7,17,25,44]. In both matrices, barn-laid eggs displayed higher viscosities than their free-range counterparts. Notably, the large differences observed between egg white and yolk under SAOS conditions at 5 °C were substantially reduced under steady shear, particularly at low shear rates (0.01 s^−1^), where the viscosity values of each egg white–yolk pair became much closer.

Flow curves confirmed markedly higher apparent viscosities in RW2 compared with RW1 across all investigated shear rates (Table 6), with statistically significant differences at 0.1, 10, and 50 s^−1^ (all *p* < 0.001). The power-law consistency index *K* was likewise significantly higher in RW2 (*p* < 0.001), whereas the flow index *n* did not differ between groups (*p* = 0.199), suggesting that both egg whites shared a similar degree of shear-thinning despite their distinct viscosity magnitudes. These differences are consistent with the structural role of the ovomucin–ovalbumin network, whose integrity and concentration strongly influence egg white viscosity [1,7,25,43]. Variations in protein composition, pH and ionic strength between production systems may further modulate network connectivity and flow resistance [17,30,44].

For yolk samples (Table 6), RY2 exhibited significantly higher viscosities and *K* values than RY1 at all tested shear rates (all *p* < 0.01), while n remained statistically similar between groups. These results collectively show that barn-laid eggs (RW2, RY2) tend to form more resistant flow structures under steady shear, whereas free-range samples (RW1, RY1) display lower flow resistance but maintain comparable shear-thinning behavior. The higher viscosities and consistency indices observed in RY2 align with established relationships between yolk flow resistance and the density and connectivity of lipoprotein granules, particularly LDL-rich plasma domains and granule-associated HDL/phosvitin complexes [3,9,18,19]. Environmental and dietary factors associated with housing systems may influence lipoprotein composition and aggregation, thereby modulating yolk rheology [21,23].

The similar flow indices across groups suggest that these structural differences affect viscosity magnitude rather than the underlying shear-thinning mechanism, consistent with the behavior of protein-rich dispersions and weakly aggregated lipoprotein networks [17,19,44].

#### 3.6.2. Three Step Shear Rate Tests

The thixotropic behavior was further examined using a three-interval step test (Figure 6b). During the first interval (low shear rate), the apparent viscosity (*ηₐ*) increased with time in both egg white and yolk, with a markedly stronger time-dependent thickening in egg white. This behavior is consistent with the lower initial viscosities and lower consistency indices (*K*) of RW1 and RW2 (Table 6), which may facilitate the rapid formation of weak, transient protein networks under low shear. Such time-dependent structuring is compatible with flexible, surface-active protein systems capable of forming reversible intermolecular associations [5,17,25].

Under high shear (second interval), *ηₐ* dropped sharply for all samples and showed minimal time dependence, consistent with fast structural breakdown. Viscosity recovery was calculated as the ratio between *ηₐ* at the end of the third interval (660 s) and that at the end of the first interval (30 s). RW1 exhibited significantly higher recovery than RW2 (*p* = 0.007), while RY1 also recovered significantly more than RY2 (*p* < 0.01), suggesting production-system-dependent differences in structural rebuildability.

A striking feature of the egg white samples is their over-recovery (Figure 6b), with final viscosities exceeding their initial values (228% for RW1 and 164% for RW2). This phenomenon is consistent with the exceptional ability of egg white proteins to reorganize into new, more cohesive networks after shear removal. Such behavior has been associated with highly surface-active, flexible protein systems and aligns with the well-known foaming ability of raw egg white, where rapid interfacial adsorption and network reorganization are thought to contribute to foam stabilization [5,22,30].

It is worth noting that the recovery curve of RW2 shows a less smooth, more irregular profile during the third interval (Figure 6b). Although the origin of this behavior cannot be determined from the present data, such fluctuations are compatible with intermittent restructuring events in weakly connected protein networks, where local breakdown and rebuilding may occur simultaneously under low shear [17,43].

In yolks, the lower recovery of RY2 compared with RY1 suggests that barn-laid yolks form more resistant but less rebuildable flow structures. This behavior is consistent with the dense packing and strong interactions among LDL, HDL and phosphoproteins in yolk granules and plasma, which govern both flow resistance and thixotropic recovery [3,9,18,19]. Free-range yolks (RY1), with lower viscosity and weaker initial structure, exhibit greater structural reversibility, consistent with more labile lipoprotein interactions [21,23].

Overall, these results suggest that barn-laid eggs develop more resistant flow structures but show reduced ability to rebuild after shear, whereas free-range samples exhibit lower viscosity but greater structural reversibility. This pattern aligns with the behavior of protein-rich versus lipoprotein-rich dispersions, where network density and interaction strength govern both steady-shear resistance and thixotropic recovery [22,24]. As described by Steffe [22], systems with more extensive protein–protein interactions form denser networks that resist deformation but recover more slowly once shear is removed. Similarly, Wang et al. [24] demonstrated that stronger intermolecular forces within protein-based gels enhance flow resistance while limiting structural rebuilding. These mechanisms are compatible with the contrasting rheological profiles observed between barn and free-range yolks.

### 3.7. Fatty Acids (FA) Profile of Raw Egg Yolks

The FA profile of raw yolks differed between free-range and barn eggs for several major components (Table 7).

Free-range yolks contained significantly higher levels of C14:0, C16:1n7, C18:1n7 and C18:3n3, whereas barn yolks showed higher concentrations of C20:4n6. In contrast, no significant differences were observed for C16:0, C18:0, C18:2n6 or C24:1n9, nor for total FA content. Oleic acid (C18:1n9) tended to be higher in free-range yolks, although the variability observed in barn samples prevented this difference from reaching statistical significance. These differences are likely associated with production-associated factors—particularly feeding regimes—rather than the housing system alone, as diet composition is known to influence yolk FA profiles. Overall, the results suggest that production conditions influence specific fatty acids, while the total lipid content remains comparable between groups. When FAs were grouped by saturation class, no significant differences were detected in total SFAs, MUFAs, or PUFAs. However, MUFA levels showed a clear trend toward higher values in free-range yolks. This pattern aligns partially with previous studies reporting that differences in yolk FA profiles are largely driven by dietary composition, which often differs between free-range and barn housing systems due to access to diverse foraging resources and variations in feed formulation [10]. The higher levels of certain MUFAs and n-3 PUFAs observed in free-range samples are compatible with this tendency, although the overall SFA/MUFA/PUFA distribution remained broadly similar between systems. Such variability is consistent with previous reports showing that diet, breed and flock management can modulate yolk composition, and that these factors often differ between production systems in commercial settings. As a result, some of the differences observed here may arise from production-associated variables rather than from housing conditions alone.

From a structural perspective, yolk lipids are organized within LDL, HDL, and granule fractions, each contributing differently to functional properties such as emulsification, viscosity, and thermal behavior [45]. Even modest shifts in the proportion of unsaturated FAs—such as the higher palmitoleic and vaccenic acids in free-range yolks—may be associated with changes in the fluidity of the LDL-rich plasma phase, potentially affecting yolk rheology upon heating. Conversely, the higher arachidonic acid content in barn yolks is also consistent with differences in dietary precursors supplied in feeding regimes characteristic of barn housing systems, rather than with the housing system itself. Finally, because yolk structural organization modulates lipid digestibility [9], the specific differences observed here—although subtle at the level of total lipid classes—could still have implications for lipid accessibility and nutritional quality. Taken together, the results suggest that the production system influences individual fatty acids more strongly than the overall saturation profile, with potential consequences for both technological and nutritional attributes of the yolk.

### 3.8. SAOS Measurements of Grilled Egg Whites and Yolks

#### 3.8.1. Stress Sweep Tests

**Figure 7:**
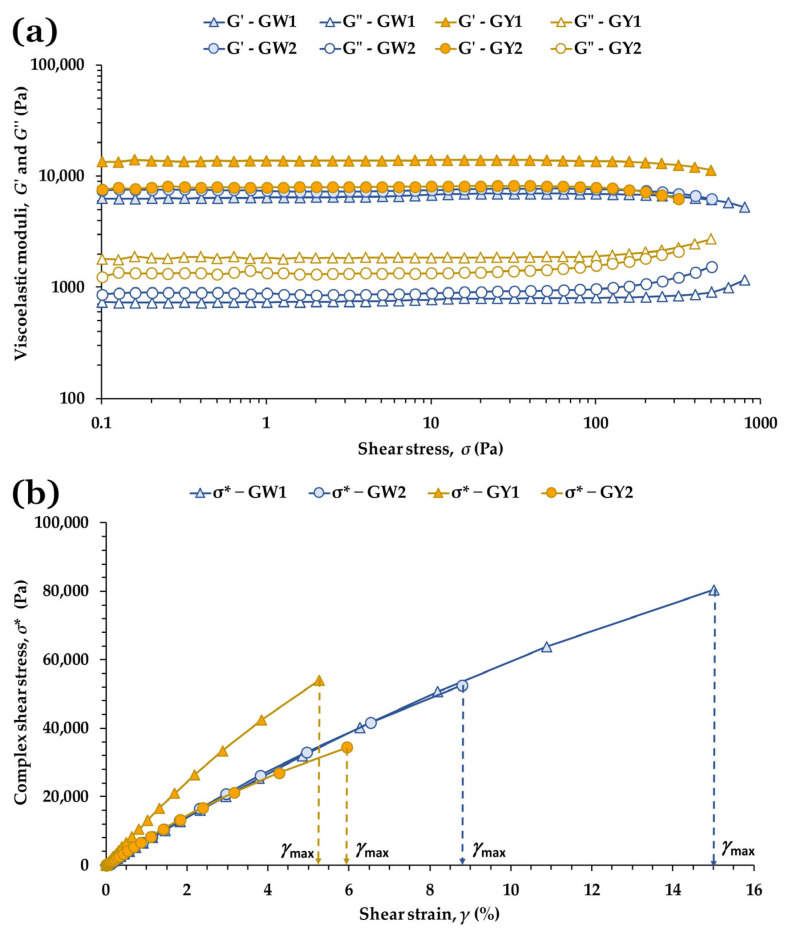
(**a**) Storage (*G*′) and loss (*G*″) moduli of grilled egg white (GW1 = free-range, GW2 = barn-laid) and yolk (GY1 = free-range, GY2 = barn-laid) as a function of shear stress at 25 °C. The linear viscoelastic region (LVR) corresponds to the plateau of *G*′ before its deviation at the critical stress (*σ*_max_). (**b**) Complex shear stress (σ*) as a function of strain (*γ*) for grilled egg white (GW1, GW2) and yolk (GY1, GY2) at 25 °C. Dashed vertical lines indicate the critical strain (*γ*_max_) for each sample.

Figure 7a shows the LVR at 25 °C for grilled egg whites and yolks from free-range (1) and barn (2) production systems. The pronounced differences previously observed between raw egg white and yolk (Figure 2) disappear after heating: all four samples display a clearly solid-like response, with *G*′ ≫ *G*″ throughout the LVR. Although GY1 still exhibits a higher *G*′ than GY2, the *G*′ curves of GW2 and GY2 nearly overlap, indicating that thermal gelation tends to homogenize the mechanical behavior of both matrices.

Heat treatment above 60–65 °C is known to denature the structure of egg-white proteins. Albumin, ovotransferrin and lysozyme are the main proteins involved in the heat-induced formation of egg-white gel [48]. As these proteins unfold, the denatured molecules aggregate through hydrophobic interactions and sulfhydryl–disulfide (SH–SS) reactions [49]. With continued cross-linking, these aggregates can evolve into an ordered three-dimensional network that becomes visible macroscopically as a firm, opaque gel. This transformation occurs in three coordinated stages: an initial increase in turbidity caused by the formation of spherical aggregates via hydrophobic interactions; a subsequent stiffening of these aggregates through SH–SS reactions; and finally, a sharp rise in elasticity during cooling, driven by rapid hydrogen-bond formation [50].

In turn, heat-induced denaturation of egg-yolk proteins occurs above 60 °C, leading to rapid protein aggregation and the formation of a thermally irreversible gel [51]. During heating, egg yolk transitions from a sol state (*G*″ > *G*′) to a solid-like structure (*G*′ > *G*″). Its native organization includes an interfacial protein membrane that separates the protein and lipid phases. Thermal denaturation reduces protein solubility but markedly increases protein concentration at this interface, an effect that becomes more pronounced as the degree of denaturation rises [52]. This enrichment of interfacial proteins enhances yolk gel formation and is considered to contribute importantly to modifying yolk interactions [13]. Because egg yolk is a natural protein–lipid supramolecular assembly, its thermal gelation is thought to depend on the combined contributions of multiple components [51]. As in the raw samples, the departure from linearity was also examined through the evolution of the complex shear stress (σ*) with strain (*γ*) (Figure 7b), providing a complementary view of structural breakdown and confirming the critical parameters extracted from the stress sweeps.

Table 8 summarizes the rheological parameters defining the LVR. All samples showed tan *δ* ≪ 1, consistent with strong gel networks formed through extensive protein denaturation and aggregation during heating [4,25]. In egg whites, GW1 exhibited significantly higher *σ*_max_ and *γ*_max_ values than GW2 (*p* < 0.001), consistent with greater resistance and deformability before leaving the LVR. Its lower tan *δ* (*p* = 0.020) further reflects a more elastic structure. Although GW2 showed a higher gel strength parameter *a* (*p* = 0.004), GW1 displayed markedly higher *b* and toughness *E* (*p* < 0.001), suggesting that GW1 forms a gel capable of absorbing substantially more energy before failure (Figure 7b). This combination of higher extensibility and toughness aligns with compositional differences between production systems that influence the stiffness and resilience of thermally induced protein gels [17,30].

For yolks, GY1 showed significantly higher *σ*_max_, *a* and *E* values than GY2 (all *p* < 0.001), together with a lower tan *δ* (*p* = 0.0002), consistent with a stronger and more elastic gel network. In contrast, *γ*_max_ did not differ significantly between yolks (*p* = 0.053), suggesting similar deformation thresholds before structural failure. The presence of lipids and lipoproteins adds complexity to yolk gelation; upon heating, LDL denature and interact with proteins, modulating network density and elasticity [2,6,18,45]. The higher *σ*_max_ and *E* values in GY1 are compatible with free-range yolks form more integrated protein–lipid networks, whereas the higher tan *δ* in GY2 reflects a softer, more dissipative structure (Table 8).

Overall, thermal treatment largely eliminates the rheological differences between raw whites and yolks, as both matrices converge toward a *G*′-dominated solid-like behavior. However, production-system effects persist after cooking: free-range samples consistently tend to form gels that are more elastic, more resistant and more energy absorbing, whereas barn samples tend to form stiffer but less cohesive networks.

#### 3.8.2. Frequency Sweep Tests

**Figure 8:**
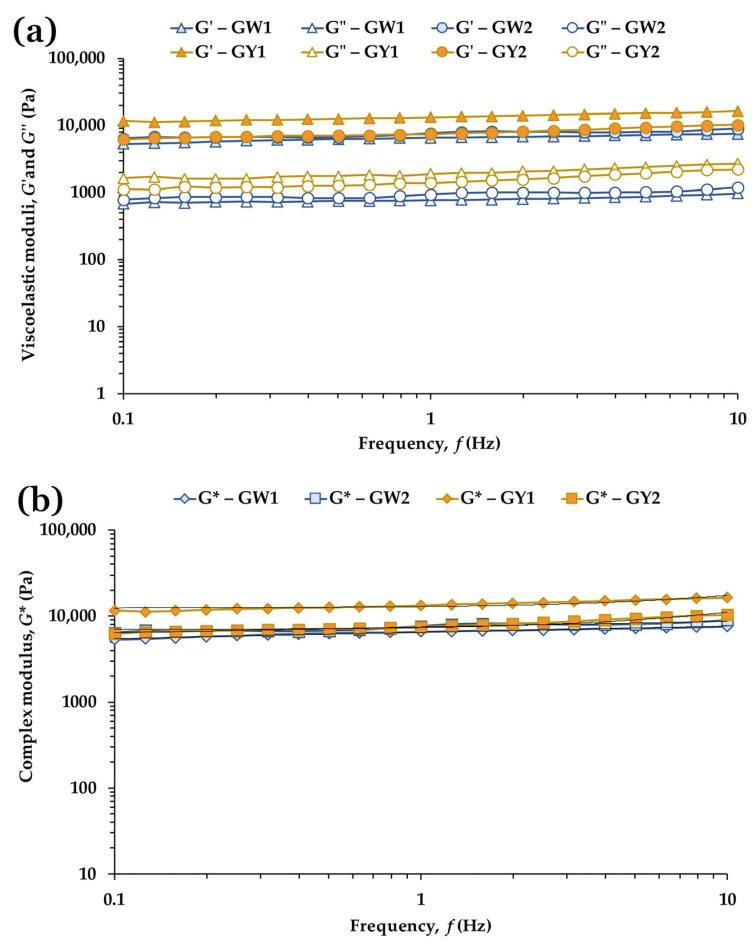
(**a**) Storage (*G*′) and loss (*G*″) moduli of grilled egg white (GW1 = free-range, GW2 = barn-laid) and yolk (GY1 = free-range, GY2 = barn-laid) as a function of frequency at 25 °C. (**b**) Complex modulus (G*) of grilled egg white (GW1, GW2) and yolk (GY1, GY2) samples over the same frequency range.

Figure 8a shows the mechanical spectra of the grilled egg whites and yolks from free-range and barn production systems. In all cases, *G*′ remained higher than *G*″ across the entire frequency range, consistent with the dominance of the elastic component in the thermally induced gels. The overall shape of the spectra was similar for whites and yolks, although production system effects were still evident. In the free-range samples, yolks exhibited higher *G*′ values than whites, whereas in the barn samples the elastic moduli of yolk and white were nearly superimposed. These differences are likely associated with compositional and structural factors associated with the feeding regimes characteristic of each system: free-range yolks contain slightly higher proportions of unsaturated fatty acids and show a more compact supramolecular organization, which may contribute to stronger gel networks upon heating, while the more uniform lipid profile of barn yolks is compatible with gels with mechanical properties closer to those of the corresponding whites.

The quantitative parameters derived from the frequency sweeps are presented in Table 9. In egg whites, the barn sample (GW2) showed significantly higher *G*′ (*p* = 0.008), *G*″ (*p* = 0.021) and complex viscosity η* (*p* = 0.008) than the free-range sample (GW1), consistent with a mechanically stronger and more viscous gel. The weak-gel parameter *A* was also significantly higher in GW2 (*p* = 0.0047), suggesting more intense intermolecular interactions within the heat-set protein network. In contrast, tan *δ* and the frequency exponent *z* did not differ significantly (*p* > 0.17 and *p* > 0.40), indicating comparable damping behavior and frequency dependence. Thus, although barn egg whites form a stiffer gel, the underlying viscoelastic character of the network remains similar across production systems. This behavior is consistent with the thermal denaturation and aggregation of ovalbumin, ovotransferrin and ovomucin, which form interconnected but deformable networks dominated by non-covalent interactions [4,25,43,53].

For yolks (Table 9), the free-range sample (GY1) exhibited significantly higher *G*′, *G*″, η* and *A* values than GY2 (all *p* < 0.01), consistent with a more elastic, viscous and cohesive gel structure. These differences are likely associated with compositional and structural variations associated with the feeding regimes characteristic of each system: the slightly higher proportion of unsaturated fatty acids and the more compact supramolecular organization observed in free-range yolks may contribute to stronger protein–lipid interactions during heating, resulting in firmer and more cohesive gels than those formed by barn-fed hens. Tan *δ* also differed significantly (*p* ≈ 0.03), with lower values in GY1, indicating a more elastic network. As in the whites, *z* did not differ significantly (*p* ≈ 0.14), suggesting that both yolks share similar frequency sensitivity despite differences in gel strength. The higher *A* value in GY1 could be compatible with a denser and more interconnected protein–lipid network, in line with the known behavior of yolk lipoproteins during heating, where LDL denature and interact with granule-associated proteins to form cohesive, fractal-like structures [2,6,18,45,54].

Both grilled egg whites and yolks showed viscoelastic behavior compatible with the weak-gel model (Figure 8b), with G* increasing slightly with frequency and maintaining a clear predominance of the elastic component. This response is consistent with the formation of interconnected but deformable networks characteristic of heat-set protein-based gels dominated by non-covalent interactions [25,44].

Finally, the standard deviations of the rheological parameters (Table 9) were consistently higher in the grilled samples than in their raw counterparts (Table 4). This increased variability is likely associated with the heterogeneity introduced during thermal processing, including non-uniform protein denaturation, water redistribution and local structural rearrangements. Additional factors such as heating gradients, surface contact during griddle cooking and slight differences in sample geometry may further contribute to the broader dispersion observed in the mechanical responses of the cooked samples.

### 3.9. Steady Shear Measurements of Grilled Egg Whites and Yolks

**Figure 9:**
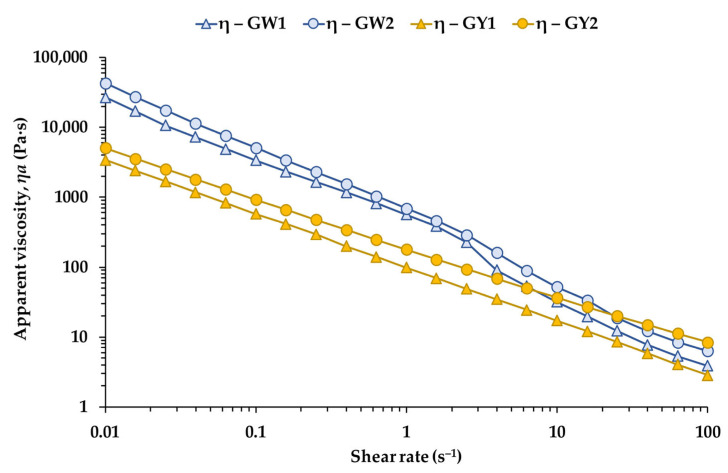
Apparent viscosity (*ηₐ*) of grilled egg white (GW1 = free-range, GW2 = barn-laid) and yolk (GY1 = free-range, GY2 = barn-laid) as a function of shear rate at 25 °C.

Figure 9 shows the flow curves of gelled egg white and yolk from free-range and barn eggs at 25 °C. As in the raw samples, all matrices exhibited clear shear-thinning behavior. However, thermal gelation appeared to reverse the relative viscosities of the components: cooked egg white became more viscous than yolk within each production system. Viscosity differences between matrices diminished at high shear rates, where GY2 even surpassed GW2. Although GW1 showed a slight deviation from linearity at intermediate shear rates, its fit to the power-law model remained excellent (*R*^2^ > 0.990; Table 10).

Table 10 summarizes the steady-shear parameters. All viscosity values differed significantly between production systems. In egg white, GW2 exhibited higher viscosities than GW1 at all shear rates (*p* ≤ 0.0006), consistent with a more structured and resistant gel network. A similar pattern was observed in yolk, where GY2 showed markedly higher viscosities than GY1 (*p* < 0.001), suggesting that the production system strongly influenced the development of the yolk matrix during heating. These differences are consistent with reports showing that protein composition, mineral content and pH—factors affected by hen housing—modulate gel strength and water-holding capacity after thermal treatment [10,13].

At 50 s^−1^, a shear rate commonly used in texture and dysphagia assessments [37,38], egg white and yolk showed comparable viscosities within each egg type (GW1 vs. GY1: 6.64 vs. 5.16 Pa·s; GW2 vs. GY2: 10.56 vs. 13.30 Pa·s). This convergence is compatible with both matrices behaving similarly under intense deformation, despite structural differences at low shear.

The consistency index *K* also differed significantly between production systems, with higher values in GW2 and GY2 (*p* ≤ 0.0005), consistent with denser and more cohesive gel networks. In cooked egg white, *n* values were extremely low (0.018–0.022), approaching the limiting case where shear stress becomes nearly independent of shear rate. Such near-zero *n* values are compatible with minimal structural reorganization under shear, consistent with rigid, heat-set protein networks formed through extensive denaturation and aggregation [53]. The small but significant difference between GW1 and GW2 (*p* = 0.021) suggests that barn-derived egg white was marginally more responsive to deformation.

In yolk, *n* values were significantly higher in GY2 than in GY1 (*p* = 0.0003), consistent with a slightly less pronounced shear-thinning behavior. These values are consistent with the heterogeneous, lipid-rich microstructure of yolk gels, which may reorganize more readily under shear than the highly protein-dense egg white gels [47]. Across both matrices, barn-housed eggs (GW2, GY2) consistently produced gels with higher viscosity, higher *K* and slightly higher *n*, suggesting firmer and more shear-dependent structures.

From a technological perspective, these rheological differences may be relevant for culinary and industrial applications. The higher viscosity and structural robustness of gels from barn-housed eggs may be advantageous for preparations requiring mechanical stability, whereas the softer, more deformable gels from free-range eggs may be advantageous in applications demanding spreadability or rapid breakdown during mastication. Extending this rheological characterization to other cooking methods (boiling, steaming, frying, baking, sous-vide) would provide further insight into how thermal and mechanical conditions shape egg-based gel structures.

### 3.10. Brightfield and Epifluorescence Microscopy of Raw and Grilled Egg Whites and Yolks

**Figure 10:**
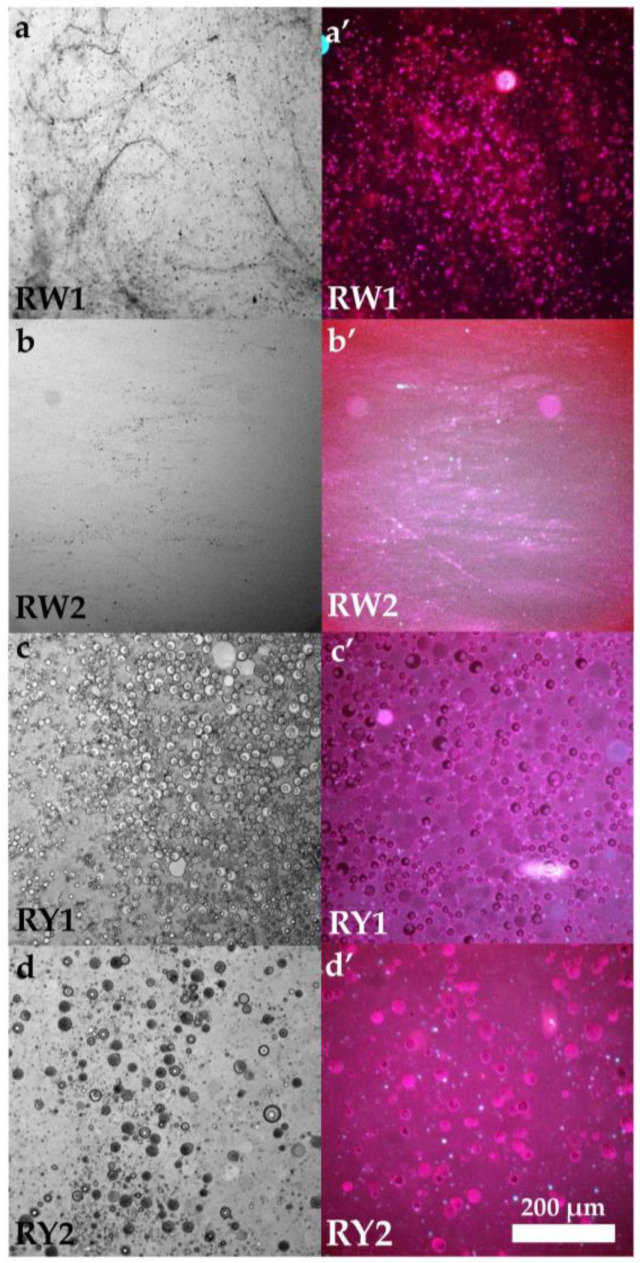
Raw egg white brightfield microscopy (**a**,**b**) and epifluorescence (**a′**,**b′**) photographs, and raw egg yolk brightfield microscopy (**c**,**d**) and epifluorescence (**c′**,**d′**) photographs. RW1 = free-range egg white; RW2 = barn-laid egg white; RY1 = free-range egg yolk; RY2 = barn-laid egg yolk.

The microstructures of the RW1 and RW2 networks, obtained through brightfield (a, b) and epifluorescence (a′, b′) microscopy, are shown in Figure 10. The RW1 microstructure (a, a′) exhibited a well-defined three-dimensional protein network, consistent with a denser and more compact egg white structure than that observed in RW2 (b, b′), with a higher number of entangled protein polymer chains. Overall, RW1 appeared to display greater egg white gel aggregation.

**Figure 11:**
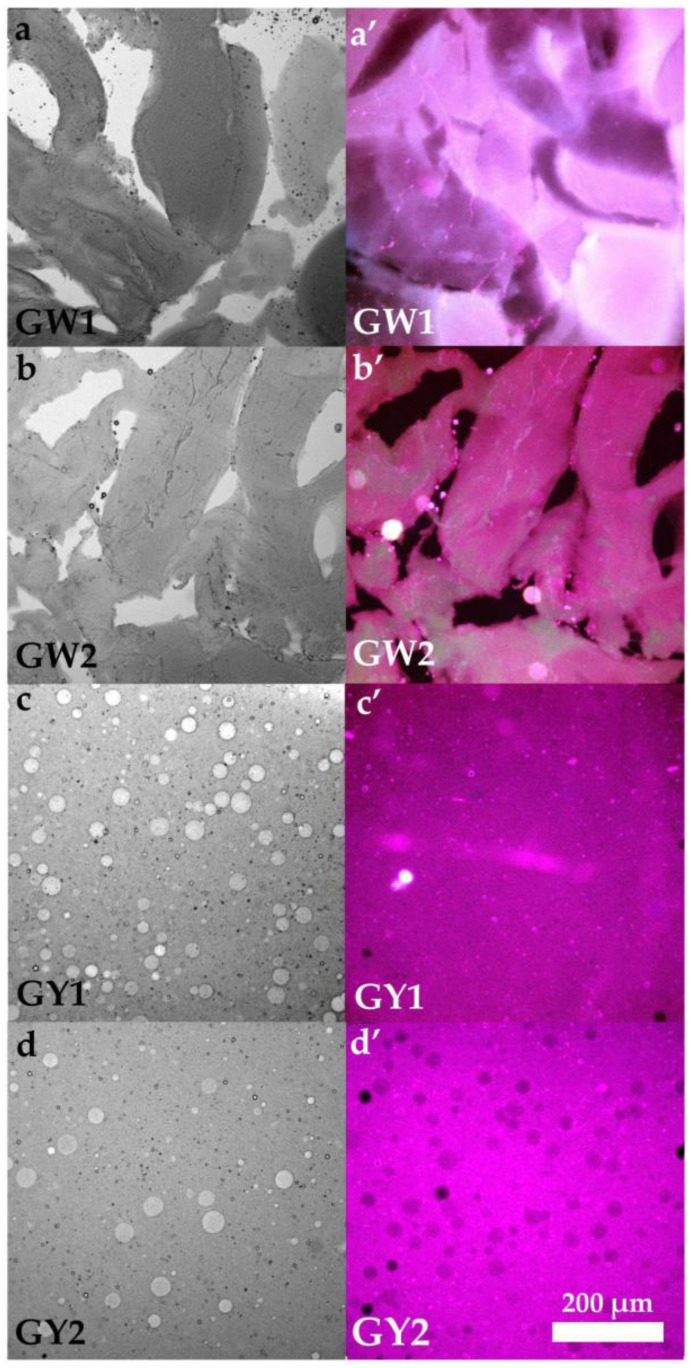
Grilled egg white brightfield microscopy (**a**,**b**) and epifluorescence (**a′**,**b′**) photographs, and grilled egg yolk brightfield microscopy (**c**,**d**) and epifluorescence (**c′**,**d′**) photographs. GW1 = free-range grilled egg white; GW2 = barn-laid grilled egg white; GY1 = free-range grilled egg yolk; GY2 = barn-laid grilled egg yolk.

Similarly, the microstructures of the RY1 and RY2 networks, obtained through brightfield (c, d) and epifluorescence (c′, d′) microscopy, are shown in Figure 11. Spherical particles corresponding to non-soluble protein aggregates (granules) suspended in a fluid phase (plasma) can be observed, as previously described by Anton et al. [3]. The RY1 network (c, c′) contained a higher number of granules, consistent with a more compact structure than that observed in RY2 (d, d′).

The images corresponding to the microstructures of GW1 (a, a′) and GW2 (b, b′) (Figure 11) show similar networks characteristic of elastic and resistant gels. This suggests that thermal treatment eliminates the differences initially observed between fresh egg whites, as also evidenced by the rheological measurements.

Regarding GY1 (c, c′) and GY2 (d, d′) (Figure 11), both samples exhibited similar networks; however, GY1 showed a greater number of aggregates and of larger size compared with GY2.

A divergence was observed between microstructural features and rheological behavior in the egg whites. RW1 exhibited significantly higher HU than RW2, consistent with superior egg white quality and a larger proportion of thick, viscous egg white. This agrees with microscopy, which showed a denser and more interconnected protein network in RW1 (Figure 10a,a′), and with their lower critical loss factor (tan *δ* = 0.436), reflecting a more viscoelastic, gel-like character. However, the rheological parameters defining the linear viscoelastic region (Table 3) revealed that this apparently stronger network was mechanically more fragile, since RW1 displayed markedly lower *σ*_max_ and *γ*_max_, as well as a much lower toughness (*E* value) within the LVR. Although RW1 appeared more compact under microscopy, compactness does not necessarily imply higher mechanical strength. A dense network may be less cross-linked or less cohesive, leading to lower rigidity and toughness despite its visual compactness. Rheology captures the ability of the matrix to resist deformation, which depends not only on compactness but also on connectivity and energy-dissipating capacity. In contrast, RW2, despite their lower HU and less compact microstructure (Figure 10b,b′), withstood substantially higher stress and strain, and exhibited an order-of-magnitude greater toughness (Table 3). These results suggest that the free-range egg white network, although visually denser and more elastic, fails at much lower deformation, whereas barn egg whites form a less viscoelastic but far more resilient and energy-absorbing structure. Such differences may be associated with compositional and structural variations associated with the feeding regimes characteristic of each system: diet-induced changes in the relative proportions of key egg white proteins (such as ovalbumin, ovotransferrin and ovomucin), together with differences in ionic balance and protein cross-linking, could contribute to networks that could be either denser but mechanically fragile, as in free-range samples, or less compact but more deformable and resistant, as observed in barn eggs. Thus, microstructure, HU, and rheology describe complementary facets of the egg white network: free-range eggs show higher structural integrity at rest, while barn eggs exhibit superior mechanical robustness under deformation.

In contrast to the behavior observed in the egg whites, yolk microstructure and rheology were largely consistent between production systems. Microscopy of RY1 (Figure 10c,c′) revealed a markedly denser and more continuous network, together with a higher concentration of spherical structures that are likely associated with yolk granules rich in high-density lipoproteins (HDL) and phosvitin [3]. This more compact microstructure is compatible with the higher critical shear stress and gel strength *a* observed for RY1 (Table 3). Although RY1 exhibited a lower critical strain, indicating a somewhat more brittle network, the total energy stored was similar for both systems. Overall, these results support the interpretation that free-range yolks could form a stronger, more structured network, while barn yolks display a slightly more deformable but mechanically weaker structure.

As this work was conceived as a technological characterization of commercially available eggs, farm-level variables such as diet composition, breed or environmental conditions could not be controlled or independently assessed. Consequently, the differences observed between free-range and barn-laid eggs should be interpreted as reflecting the natural variability present among products marketed under these two commercial categories, rather than as isolated causal effects of specific on-farm practices. From a food technology perspective, this interpretation is particularly relevant, as it provides insight into the functional behavior and processing performance of eggs as they are encountered by consumers and used in culinary and industrial applications.

## 4. Conclusions

This study shows that commercially available eggs labelled as free-range and barn-laid display consistent differences in the behavior of both raw and cooked egg matrices. These differences reflect variations in structural organization and composition of egg white and yolk, as indicated by traits such as HU and yolk redness (a*), and by the contrasting rheological responses of the two matrices. Barn-laid eggs showed a more cohesive and deformation-resistant egg-white network, whereas free-range eggs exhibited a stronger viscoelastic response in the yolk. After cooking, both matrices formed strong elastic gels, although free-range eggs produced more elastic and reversible networks, while barn-laid eggs generated firmer, less rebuildable structures. These functional contrasts may guide the selection of egg types for specific technological applications.

Importantly, the eggs analyzed were obtained from retail sources, and key variables such as hen diet, genotype, flock management, and exact egg age were not controlled. Therefore, the observed differences cannot be attributed solely to the production system and should be interpreted as market-level comparisons rather than controlled experimental evidence. Future studies using standardized flocks and controlled conditions are needed to isolate the contribution of individual biological and management factors.

## Figures and Tables

**Table 1:** Physical measurements of eggs from free-range and barn-laid production systems.

Variable	Free-Range Production	Barn-Laid Production
Whole egg weight (g)	64.06 ± 4.94 ^A^	67.64 ± 2.95 ^A^
Egg yolk weight (g)	18.11 ± 2.06 ^A^	18.48 ± 1.59 ^A^
Egg white weight (g)	35.25 ± 3.20 ^B^	38.56 ± 2.99 ^A^
Egg shell weight (g)	8.42 ± 0.741 ^B^	9.21 ± 0.778 ^A^
Equatorial diameter (mm)	44.25 ± 1.45 ^A^	44.99 ± 0.907 ^A^
Egg height (mm)	57.96 ± 2.24 ^A^	58.31 ± 1.58 ^A^

Values are expressed as the mean (*n* = 20) ± standard deviation. Different superscript letters (A, B) within each row indicate significant differences between production systems (*p* < 0.05).

**Table 2:** Significant Pearson correlations between whole-egg weight, internal components, and morphometric measurements in free-range and barn-laid eggs.

Correlation Pair	Free-Range (*r*)	Barn-Laid (*r*)
Whole egg weight vs. Egg white	0.907 **	0.704 *
Egg height vs. Yolk	0.760 **	-
Whole egg weight vs. Egg height	0.731 *	-
Whole egg weight vs. Equatorial diameter	0.818 **	0.684 *

Only statistically significant relationships are shown; non-significant correlations are indicated with “-”. Asterisks denote significance levels (* *p* < 0.05; ** *p* < 0.01).

**Table 3:** Critical rheological parameters defining the linear viscoelastic region (LVR) of raw egg white and yolk at 5 °C from free-range (1) and barn-laid (2) production systems.

Sample	*σ*_max_ (Pa)	*γ*_max_ (%)	tan *δ* (–)	*a* (Pa)	*b* (Pa)	*R*^2^ (Equation (2))	*E* (J/m^3^)
RW1	0.132 ± 0.002 ^B^	212 ± 15.9 ^B^	0.467 ± 0.046 ^B^	0.062 ± 0.004 ^B^	0.078 ± 0.007 ^A^	1.00	6602 ± 540 ^B^
RW2	0.554 ± 0.018 ^A^	768 ± 12.6 ^A^	0.557 ± 0.027 ^A^	0.074 ± 0.005 ^A^	−0.831 ± 0.076 ^B^	1.00	113,439 ± 3900 ^A^
RY1	1.78 ± 0.002 ^A^	5.57 ± 0.924 ^B^	4.77 ± 0.012 ^A^	37.0 ± 2.40 ^A^	2.52 ± 0.241 ^A^	0.998	2421 ± 260 ^A^
RY2	1.27 ± 0.001 ^B^	7.98 ± 0.195 ^A^	3.54 ± 0.229 ^B^	16.5 ± 0.450 ^B^	2.66 ± 0.140 ^A^	0.995	2367 ± 62.0 ^A^

Mean values (*n* = 3) ± standard deviation. *σ*_max_, critical value of shear stress; *γ*_max_, critical value of shear strain; tan *δ*, critical value of the loss factor (tan *δ* = *G*″/*G*′). *a*, gel strength; *b*, initial stress (*σ*_0_ at *γ*_0_); *R*^2^: coefficient of determination for Equation (2). *E*, total energy involved in the linear deformation (toughness). Different superscript letters (A, B) within each pair of samples (RW1 = free-range egg white, RW2 = barn-laid egg white; RY1 = free-range yolk, RY2 = barn-laid yolk) in the same column indicate significant differences (*p* < 0.05).

**Table 4:** Mechanical spectra and weak-gel model parameters of raw egg white and yolk at 5 °C from free-range (1) and barn-laid (2) production systems.

Sample	*G*′ (Pa)	*G*″ (Pa)	tan *δ* (–)	η* (Pa·s)	*A* (Pa·s^1/^*^z^*)	*z* (–)	*R*^2^ (Equation (4))
RW1	0.032 ± 0.001 ^B^	0.023 ± 0.000 ^B^	0.720 ± 0.020 ^A^	0.064 ± 0.001 ^B^	-	-	-
RW2	0.066 ± 0.003 ^A^	0.041 ± 0.000 ^A^	0.623 ± 0.029 ^B^	0.124 ± 0.004 ^A^	-	-	-
RY1	6.71 ± 0.230 ^A^	31.6 ± 1.28 ^A^	4.71 ± 0.135 ^A^	5.14 ± 0.205 ^A^	32.8 ± 0.565 ^A^	1.12 ± 0.008 ^B^	1.00
RY2	5.10 ± 0.305 ^B^	14.4 ± 1.00 ^B^	2.82 ± 0.231 ^B^	2.43 ± 0.207 ^B^	14.8 ± 0.976 ^B^	1.19 ± 0.008 ^A^	0.999

Mean values (*n* = 3) ± standard deviation. *G*′, elastic or storage modulus; *G*″, viscous or loss modulus; tan *δ*, loss factor (=*G*″/*G*′). *A* and *z*: weak-gel model parameters representing strength of the interactions and coordination number, respectively; *R*^2^: coefficient of determination for Equation (4). Different superscript letters (A, B) within each pair of samples (RW1 = free-range egg white, RW2 = barn-laid egg white; RY1 = free-range yolk, RY2 = barn-laid yolk) in the same column indicate significant differences (*p* < 0.05).

**Table 5:** Crossover temperature and viscoelastic moduli of raw egg white and yolk at 25 °C from free-range (1) and barn-laid (2) production systems.

Sample	Temperature (°C)	*G*′ (Pa)	*G*″ (Pa)
RW1	-	0.057 ± 0.006 ^B^	0.032 ± 0.002 ^B^
RW2	-	0.159 ± 0.007 ^A^	0.053 ± 0.005 ^A^
RY1	36.8 ± 1.36 ^B^	1.07 ± 0.142 ^B^	8.62 ± 0.008 ^B^
RY2	44.0 ± 1.62 ^A^	3.73 ± 0.142 ^A^	11.6 ± 1.02 ^A^

Mean values (*n* = 3) ± standard deviation. *G*′, elastic or storage modulus; *G*″, viscous or loss modulus; tan *δ*, loss factor (=*G*″/*G*′). Different superscript letters (A, B) within each pair of samples (RW1 = free-range egg white, RW2 = barn-laid egg white; RY1 = free-range yolk, RY2 = barn-laid yolk) in the same column indicate significant differences (*p* < 0.05).

**Table 6:** Steady-shear apparent viscosities (*η*_0.1_, *η*_10_, and *η*_50_) and power-law parameters (*K*, *n*) of raw egg white and yolk at 20 °C from free-range (1) and barn-laid (2) production systems.

Sample	*η*_0.1_ (Pa·s)	*η*_10_ (Pa·s)	*η*_50_ (Pa·s)	*K* (Pa·s*^n^*)	*n* (–)	*R*^2^ (Equation (5))	Recovered Viscosity (%)
RW1	0.191 ± 0.016 ^B^	0.020 ± 0.002 ^B^	0.014 ± 0.001 ^B^	0.070 ± 0.006 ^B^	0.446 ± 0.019 ^B^	0.966	228 ± 19 ^A^
RW2	0.854 ± 0.003 ^A^	0.079 ± 0.008 ^A^	0.046 ± 0.000 ^A^	0.278 ± 0.002 ^A^	0.426 ± 0.013 ^B^	0.969	164.2 ± 9.9 ^B^
RY1	1.20 ± 0.13 ^B^	0.638 ± 0.033 ^B^	0.581 ± 0.037 ^B^	0.918 ± 0.065 ^B^	0.844 ± 0.047 ^B^	0.997	79.1 ± 1.2 ^A^
RY2	2.69 ± 0.28 ^A^	1.33 ± 0.038 ^A^	1.14 ± 0.016 ^A^	1.91 ± 0.12 ^A^	0.850 ± 0.046 ^B^	0.998	39.6 ± 2.6 ^B^

Mean (*n* = 3) ± SD; *K* and *n*: consistency index and flow behavior index from power-law model; *R*^2^: coefficient of determination for Equation (5). Different superscript letters (A, B) within each pair of samples (RW1 = free-range egg white, RW2 = barn-laid egg white; RY1 = free-range yolk, RY2 = barn-laid yolk) in the same column indicate significant differences (*p* < 0.05).

**Table 7:** Major fatty acids (FA) present in raw egg yolks from free-range and barn-laid production systems (g/100 g yolk).

FA and Saturation Class	Free-Range	Barn-Laid
C14:0	0.078 ± 0.0002 ^A^	0.067 ± 0.0003 ^B^
C16:0	6.51 ± 0.025 ^A^	6.05 ± 0.333 ^A^
C18:0	2.17 ± 0.010 ^A^	2.23 ± 0.120 ^A^
∑SFA	8.76 ± 0.034 ^A^	8.34 ± 0.450 ^A^
C16:1n7	0.634 ± 0.003 ^A^	0.536 ± 0.029 ^B^
C18:1n7	0.478 ± 0.002 ^A^	0.413 ± 0.023 ^B^
C18:1n9	10.37 ± 0.049 ^A^	9.53 ± 0.517 ^A^
C24:1n9	0.204 ± 0.001 ^A^	0.209 ± 0.011 ^A^
∑MUFA	11.69 ± 0.055 ^A^	10.69 ± 0.580 ^A^
C18:2n6	6.29 ± 0.031 ^A^	6.37 ± 0.349 ^A^
C18:3n3	0.201 ± 0.001 ^A^	0.177 ± 0.010 ^B^
C20:4n6	0.529 ± 0.003 ^B^	0.677 ± 0.036 ^A^
∑PUFA	7.02 ± 0.035 ^A^	7.22 ± 0.390 ^A^
Total FA	27.46 ± 0.125 ^A^	26.25 ± 1.43 ^A^

Mean (*n* = 3) ± SD; SFA, saturated fatty acids; MUFA, monounsaturated fatty acids; PUFA, polyunsaturated fatty acids. Different superscript letters (A, B) within the same row indicate significant differences (*p* < 0.05).

**Table 8:** Critical rheological parameters defining the linear viscoelastic region (LVR) of grilled egg white and yolk at 25 °C from free-range (1) and barn-laid (2) production systems.

Sample	*σ*_max_ (Pa)	*γ*_max_ (%)	tan *δ* (–)	*a* (Pa)	*b* (Pa)	*R*^2^ (Equation (2))	*E* (J/m^3^)
GW1	804 ± 0.10 ^A^	15.01 ± 0.13 ^A^	0.222 ± 0.0070 ^B^	5739 ± 47 ^B^	683 ± 5.1 ^A^	0.992	1,511,054 ± 12,543 ^B^
GW2	507 ± 1.7 ^B^	8.80 ± 0.80 ^B^	0.250 ± 0.012 ^A^	6720 ± 63 ^A^	355 ± 31 ^B^	1.00	587,539 ± 49,304 ^A^
GY1	503 ± 0.65 ^A^	5.27 ± 0.27 ^A^	0.293 ± 0.012 ^B^	12,199 ± 54 ^A^	386 ± 23 ^A^	0.994	352,815 ± 20,720 ^A^
GY2	318 ± 0.65 ^B^	5.95 ± 0.63 ^A^	0.417 ± 0.024 ^A^	6998 ± 240 ^B^	329 ± 33 ^A^	0.991	249,844 ± 26,219 ^B^

Mean values (*n* = 3) ± standard deviation. *σ*_max_, critical value of shear stress; *γ*_max_, critical value of shear strain; tan *δ*, critical value of the loss factor (tan *δ* = *G*″/*G*′). *a*, gel strength; *b*, initial stress (*σ*_0_ at *γ*_0_); *R*^2^: coefficient of determination for Equation (2). *E*, total energy involved in the linear deformation (toughness). Different superscript letters (A, B) within each pair of samples (GW1 = free-range grilled egg white, GW2 = barn-laid grilled egg white; GY1 = free-range grilled yolk, GY2 = barn-laid grilled yolk) in the same column indicate significant differences (*p* < 0.05).

**Table 9:** Mechanical spectra and weak-gel model parameters of grilled egg white and yolk at 25 °C from free-range (1) and barn-laid (2) production systems.

Sample	*G*′ (Pa)	*G*″ (Pa)	tan *δ* (–)	η* (Pa·s)	*A* (Pa·s^1/z^)	z (–)	*R*^2^ (Equation (4))
GW1	6516 ± 330 ^B^	765 ± 28 ^B^	0.117 ± 0.0020 ^B^	1044 ± 53 ^B^	6469 ± 160 ^B^	14.0 ± 1.4 ^B^	0.906
GW2	7607 ± 154 ^A^	938 ± 130 ^A^	0.123 ± 0.014 ^B^	1220 ± 6.0 ^A^	7094 ± 150 ^A^	14.1 ± 0.08 ^B^	0.858
GY1	13,148 ± 3500 ^A^	1895 ± 370 ^A^	0.146 ± 0.011 ^B^	2115 ± 560 ^A^	11,961 ± 4400 ^A^	12.2 ± 2.8 ^B^	0.983
GY2	7408 ± 1000 ^B^	1381 ± 43 ^B^	0.188 ± 0.020 ^A^	1200 ± 110 ^B^	8083 ± 830 ^B^	8.87 ± 1.3 ^B^	0.960

Mean values (*n* = 3) ± standard deviation. *G*′, elastic or storage modulus; *G*″, viscous or loss modulus; tan *δ*, loss factor (=*G*″/*G*′). *A* and *z*: weak-gel model parameters representing strength of the interactions and coordination number, respectively; *R*^2^: coefficient of determination for Equation (4). Different superscript letters (A, B) within each pair of samples (GW1 = free-range grilled egg white, GW2 = barn-laid grilled egg white; GY1 = free-range grilled yolk, GY2 = barn-laid grilled yolk) in the same column indicate significant differences (*p* < 0.05).

**Table 10:** Steady-shear apparent viscosities (*η*_0.1_, *η*_10_, and *η*_50_) and power-law parameters (*K*, *n*) for grilled egg white and yolk at 25 °C.

Sample	*η*_0.1_ (Pa·s)	*η*_10_ (Pa·s)	*η*_50_ (Pa·s)	*K* (Pa·s*^n^*)	*n* (–)	*R*^2^ (Equation (5))
GW1	3279 ± 100 ^B^	31.14 ± 0.60 ^B^	6.64 ± 0.035 ^B^	357 ± 7.5 ^B^	0.018 ± 0.00020 ^B^	0.992
GW2	5128 ± 349 ^A^	51.87 ± 0.13 ^A^	10.56 ± 0.12 ^A^	546 ± 19 ^A^	0.022 ± 0.0040 ^A^	0.996
GY1	585 ± 5.0 ^B^	16.71 ± 0.50 ^B^	5.16 ± 0.10 ^B^	96.58 ± 3.0 ^B^	0.227 ± 0.0050 ^B^	0.999
GY2	922 ± 27 ^A^	36.62 ± 1.5 ^A^	13.30 ± 1.4 ^A^	187 ± 20 ^A^	0.285 ± 0.0039 ^A^	0.996

Mean (*n* = 3) ± SD; *K* and *n*: consistency index and flow behavior index from power-law model; *R*^2^: coefficient of determination for Equation (5). Different superscript letters (A, B) within each pair of samples (GW1 = free-range grilled egg white, GW2 = barn-laid grilled egg white; GY1 = free-range grilled yolk, GY2 = barn-laid grilled yolk) in the same column indicate significant differences (*p* < 0.05).

## Data Availability

All the raw data for this article, used in the generation of tables and figures, are available at the public repository digital.csic.es of the Spanish Research Council (CSIC) at https://doi.org/10.20350/digitalCSIC/18224.
